# Lifestyle Impacts the Oral Microbiome of Classical and Post‐Classical Societies in Italy

**DOI:** 10.1002/ajpa.70357

**Published:** 2026-09-08

**Authors:** Martina Farese, Markella Moraitou, Chenyu Jin, Adrian Forsythe, Ileana Micarelli, Tom van der Valk, Giorgio Manzi, Laura Parducci, Mary Anne Tafuri, Katerina Guschanski

**Affiliations:** ^1^ Department of Environmental Biology and Mediterranean bioArchaeological Research Advances (MAReA) Centre Sapienza University of Rome Rome Italy; ^2^ Institute of Ecology and Evolution, School of Biological Sciences, University of Edinburgh Edinburgh UK; ^3^ Centre for Palaeogenetics Stockholm Sweden; ^4^ Department of Bioinformatics and Genetics Swedish Museum of Natural History Stockholm Sweden; ^5^ Department of Zoology Stockholm University Stockholm Sweden; ^6^ Department of Ecology and Genetics, Animal Ecology, Evolutionary Biology Centre Uppsala University Uppsala Sweden; ^7^ Department of Environmental Biology Sapienza University of Rome Rome Italy; ^8^ Science for Life Laboratory Uppsala Sweden

**Keywords:** ancient DNA, bioarchaeology, dental calculus, metagenomics, Rome

## Abstract

**Objectives:**

The fall of the Roman Empire (476 CE) profoundly affected the lives of its peoples due to the political, administrative, and territorial changes that occurred. The majority of written records of the time focus on the social élite, leaving larger parts of the population understudied. Here, we employ a bioarchaeological approach to understand how differences in lifestyle may be reflected in the oral microbiome of people from different social classes living before and after the fall.

**Material and Methods:**

We analyzed shotgun sequencing data from dental calculus, the preserved oral microbiome, of 67 individuals belonging to different social classes from two Classical cemeteries (I–III century CE, Lucus Feroniae and Isola Sacra) and one post‐Classical cemetery (IV–VIII century CE, Selvicciola), all located in proximity to the city of Rome, Italy.

**Results:**

We detect significant differences in the taxonomic and functional composition of the oral microbiome between the three sites, with the rural town of Lucus Feroniae standing out compared to its two counterparts. Reliable identification of dietary items was not possible.

**Discussion:**

The distinct oral microbiome of Lucus Feroniae could reflect differences in general health and subsistence practices, in line with previously published isotopic and morphological data. Its rural position may have mitigated the cyclical food crises that affected the contemporary Isola Sacra and the later community of Selvicciola, buffering it against the nutritional stress observed in these two locations. This finding supports the temporal stability of the dental calculus microbiome while highlighting the impact of lifestyle on the oral microbial communities.

## 1 Introduction

Lifestyles in the Roman Empire and Late Antiquity have been extensively researched in the last two decades, with studies aiming to reconstruct social organizations, dietary practices, health conditions, and economic activities (Barchiesi and Scheidel 2010; Hingley 2005; Purcell 2003; Twiss 2019). The surviving written records also offer means to investigate the many facets that constituted Roman and post‐Classical societies (approximately 753 BCE–1492 CE). However, as written sources are usually dominated by the most influential societal strata (the élite), studies based on these records are often biased towards the lifestyles of the upper class who lived in central cities, overlooking the daily lives of the majority of the population. We therefore lack a comprehensive understanding of the everyday lives of people from less prominent social classes, who mainly lived outside major cities. This societal stratum was dominated by farmers, war veterans, merchants, freedmen, and slaves, who played essential roles in the economy of the times but are underrepresented in historical texts and iconography (Allison 1999; Dench 2010; Hingley 2005; Tacoma 2016). Direct analyses of skeletal material offer insights into the changes brought about by periods of major social, cultural, and political upheaval, as in the case of the fall of the Roman Empire (476 CE). The study of human remains recovered from archaeological sites around the city of Rome can help address the gap left by written records, including uncovering changes in health, diet, and living conditions in both rural and urban areas, therefore providing a more complete picture of the past.

Different techniques, such as morphological, paleopathological, isotopic and molecular analyses, have been applied to investigate the lifestyles, diets and pathologies of ancient peoples, including those from lower societal classes (Emery, Duggan, et al. 2018; Emery, Stark, et al. 2018; Manzi et al. 1991; Salvadei et al. 2001; Tafuri et al. 2018). Recently, the study of dental calculus, the mineralized form of dental plaque, has provided unique insights into ancient societies (Bravo‐Lopez et al. 2020; Eisenhofer et al. 2020; Gancz et al. 2023; Ottoni et al. 2021; Quagliariello et al. 2022; Velsko et al. 2022, 2023, 2024; Warinner et al. 2014). This mineralized deposit fossilizes on the living host, reducing post‐mortem contamination from exogenous microorganisms and allowing the preservation of endogenous DNA in a virtually unchanged form for millennia (Mann et al. 2018; Warinner et al. 2015). Dental calculus preserves the remains of oral and respiratory microbial communities, dietary elements and host‐derived molecules (Ozga et al. 2016; Velsko et al. 2022; Warinner et al. 2014).

The dental calculus microbiome appears to have been rather stable over time (within the last 8000 years), even during major cultural transitions, including the introduction of agriculture and the Industrial Revolution (Gancz et al. 2023; Ottoni et al. 2021; Velsko et al. 2023). Several studies have attempted to use the dental calculus microbiome to shed light on how communities and their lifestyles have developed. However, Ottoni et al. (2021) found that the oral microbiomes of humans living in the Paleolithic and Mesolithic periods remained stable, with only minor changes detected since the introduction of agriculture. Similarly, a comparative study spanning from the Neolithic to the Industrial Revolution found no significant changes in the dental calculus microbiome composition (Gancz et al. 2023). A strong stability was also observed when comparing foraging and highly industrialized populations, with dietary differences playing only a minor role in the composition of dental plaque in human populations with different subsistence strategies (Velsko et al. 2023). However, despite a lack of temporal changes, a distinct oral microbial community composition was recovered from dental calculus in Oceania, setting it apart from Europe, Asia and Africa (Velsko et al. 2024). Supporting this, the dental calculus microbiome composition and function of closely related gorilla (sub)species were shown to vary with host ecology, most likely reflecting different diets (Moraitou et al. 2022), despite their geographic proximity and recent divergence of only 12,000 years ago (Van Der Valk et al. 2024). This suggests that subtle but persistent differences in lifestyle and diet might be reflected in the dental calculus microbiome in humans as well. If so, this will open doors to studying ancient human communities that lack other sources of information about their lifestyles.

**FIGURE 1:**
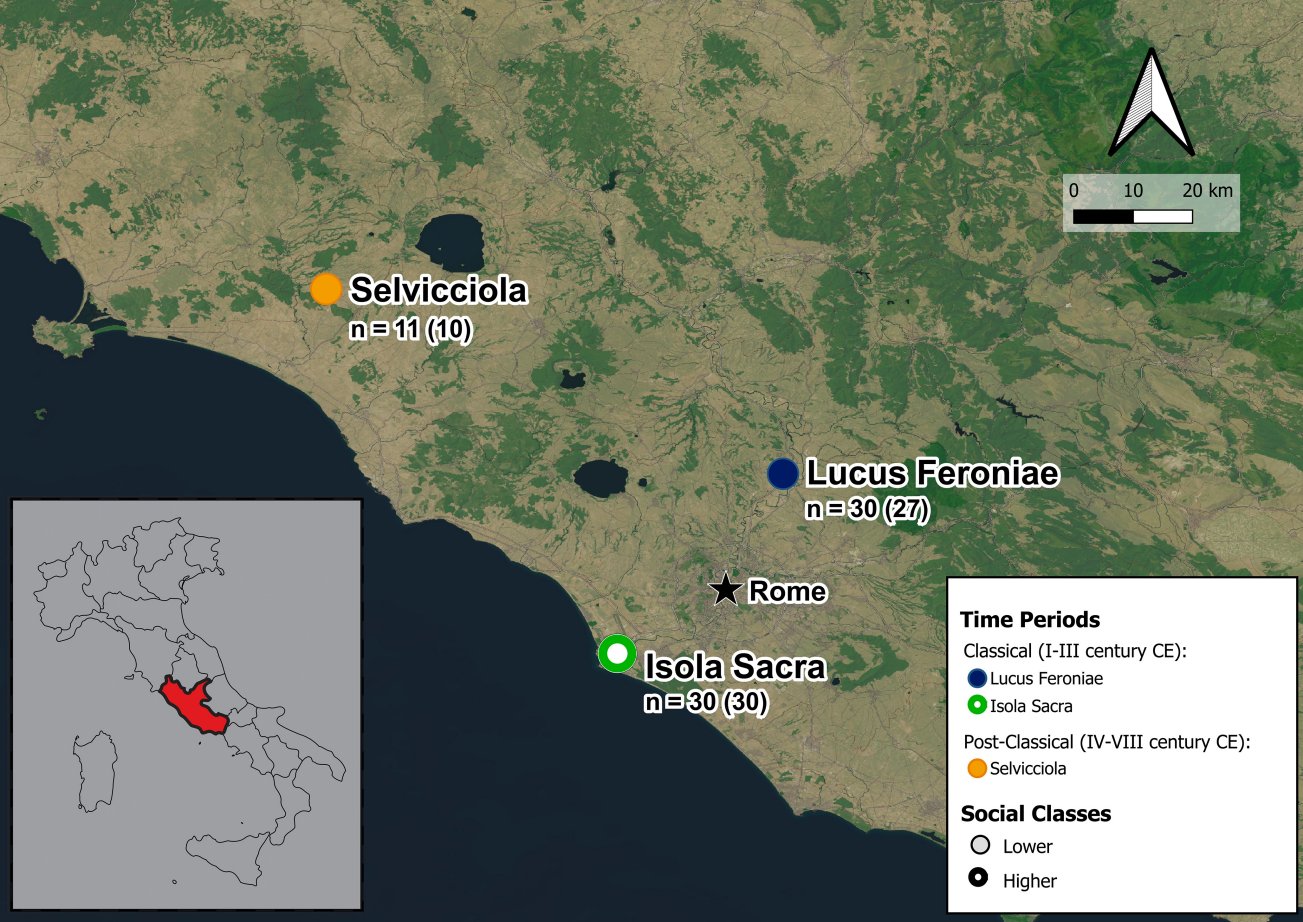
Map showing the location of the archaeological sites presented in this study, with the city of Rome displayed as a black star. Number of dental calculus samples (*n*) for each site, with number of unique individuals within brackets (see Table 1; map created with QGIS 3.36.3).

So far, only a few studies have examined human communities living in geographic proximity but differing in their lifestyles and diet (Cameron et al. 2015; Sarkar et al. 2017; Velsko et al. 2023). However, these studies focused primarily on modern populations, with a notable exception of the recent investigation of archaeological samples from Oceania (Velsko et al. 2024). Here, we aim to advance our understanding of past societies, particularly communities underrepresented in classical literature. To do so, we conducted comparative analyses of the dental calculus oral microbiome from three archaeological sites from the vicinity of the city of Rome, Italy (Figure 1). These include two contemporaneous communities that lived during the Roman Imperial period but differed in their lifestyles (Isola Sacra and Lucus Feroniae) and a community dating to the Late Antique and Longobard period (Selvicciola). By characterizing oral microbiomes, we aim to investigate how differences in socio‐economic status during the Roman Empire and the cultural transition that followed its fall impacted local human societies.

## 2 Materials and Methods

### 2.1 Archaeological Sites

#### 2.1.1 Isola Sacra

The cemetery of Isola Sacra is linked to the ancient city of Portus Romae, about 20 km west of Rome (Figure 1). It dates back to the Roman Imperial age and, as no radiocarbon dates are available, the individuals are generally attributed to the I–III century CE, based on the archaeological material recovered. The cemetery is located along the road connecting the city of Portus to the coast and the town of Ostia, the Via *Flavia*, on the so‐called “sacred island” (Baldassarre 2002; Prowse et al. 2004; Verduchi 1992). Portus was built around the port established by Emperor Claudius and expanded by Emperor Trajan between 42 and 64 CE. The importance of Portus grew during the expansion of Rome, being the main harbor of the capital of the Empire. The inhabitants of Portus included merchants, administrators and traders; however, no local aristocracy is mentioned in the written records. Dietary analysis by means of carbon and nitrogen stable isotopes, performed on a subgroup of 105 individuals buried in the cemetery of Isola Sacra, suggests a mixed diet composed of marine and terrestrial resources (Prowse et al. 2004, 2005).

#### 2.1.2 Lucus Feroniae

The cemetery is connected to the Roman town of Lucus Feroniae, 30 km northeast of Rome (Figure 1). No radiocarbon dates are available, but, as for Isola Sacra, the cemetery was dated to the I–III century CE on the basis of the archaeological material. Lucus Feroniae was a rural centre with an economy based on wheat cultivation (Gazzetti 1986). In 31 BCE, after the defeat of Marcus Antonius in Actium, Augustus assigned several areas of the town to his troops, thereby making the soldiers part of the population, alongside the locals (Stanco 1997). The general lack of grave goods inside the tombs and the presence of developmental and occupational stress markers on the skeletal remains (Manzi et al. 1989; Sperduti 1997) of several individuals suggest that the cemetery holds manual workers and people of humble origins, war veterans, *liberti* (freedmen and freedwomen), and slaves. The stable isotope data suggest that, despite being a rural town, people living in Lucus Feroniae had access to a variety of food resources, including aquatic products, as a result of both agricultural activities carried out at the site and the exploitation of the local environment (Tafuri et al. 2018).

#### 2.1.3 Selvicciola

The cemetery of Selvicciola, located near the town of Ischia di Castro (Viterbo), 90 km north of Rome (Figure 1), has been dated to between the end of the IV and the beginning of the VIII century CE (Gazzetti 1995, 1997; Incitti 1997; Micarelli 2020; Patera 2008). After the fall of the Western Roman Empire in 476 ce, the area was dominated by the Longobards, with archaeological findings also testifying to the presence of a wealthy community since the VI–VII centuries CE. Selvicciola, a Longobard site dated to the mid‐VII century CE, was built over an older Roman settlement (Patera 2009). The anthropological investigations performed on the human remains from Selvicciola suggest a lower quality of life compared to Lucus Feroniae and Isola Sacra, with a higher incidence of dental and skeletal pathologies (Manzi et al. 1999; Salvadei et al. 2001). The low variability in nitrogen stable isotope data suggests that the diet of individuals buried in Selvicciola was based on a limited selection of resources, with a stronger reliance on grains than in Lucus Feroniae (Tafuri et al. 2018).

### 2.2 Sample Collection

We collected a total of 71 dental calculus samples from 67 individuals housed in the Museum of Anthropology “Giuseppe Sergi”, part of the Sapienza University of Rome Museum Network (*Polo Museale*), from both healthy and carious teeth (Table 1). Thirty of the sampled individuals were buried in the cemetery of Isola Sacra, 27 in Lucus Feroniae and 10 in Selvicciola. Among the collected samples, 58 were from individuals without signs of dental caries (although some showed other oral pathologies, such as antemortem tooth loss, linear enamel hypoplasia, dental fractures or heavy tooth wear), six were from within the caries lesions, and seven were from healthy teeth in individuals that showed caries elsewhere in the oral cavity (Table S1). We also collected eight soil samples (seven from Lucus Feroniae and one from Selvicciola) to act as environmental controls. As no soil was deliberately sampled during the excavation, these samples were obtained from soil particles that adhered to the bones of the study individuals. We prioritized skeletal positions as far away from the cranium and the dental calculus as possible (Table S1). No soil samples were available from Isola Sacra. Sampling was carried out following procedures specific for ancient DNA sample collections (Sabin and Fellows Yates 2020; Velsko et al. 2017; Warinner et al. 2019). All samples were then transferred to Uppsala University, Sweden, for processing.

**TABLE 1:** Overview of the samples used in this study.

Cemetery	Time period	Archaeological period	Latitude (N)	Longitude (E)	N of dental calculus samples (*N* of unique individuals)	*N* of soil samples
Isola Sacra	I–III century	Classical	41.769	12.264	30 (30)	—
Lucus Feroniae	I–III century	Classical	42.130	12.597	30 (27)	7
Selvicciola	IV–VIII century	Post‐classical	42.501	11.679	11 (10)	1
Total					71 (67)	8

*Note:* See Table S1 for more details.

### 2.3 Sample Preparation and Shotgun Sequencing

All samples were processed in the dedicated ancient DNA laboratory. The calculus samples (weighing between < 5 and 20 mg) were ground with sterile micro‐pestles. Surface decontamination was performed as in Brealey et al. (2021), exposing the samples to UV light (245 nm) for 10 min, followed by a wash in 500 μL of 0.5 M EDTA pH 8 for 30 s. Both dental calculus and soil samples were extracted with a silica‐based method (Dabney et al. 2013) adapted for dental calculus, as previously described in Brealey et al. (2020). Briefly, after the wash step and centrifugation, the residual pellets were treated with 1 mL of extraction buffer (0.45 M EDTA pH 8, 0.25 mg/mL Proteinase K) and left to digest in a rotating incubator at 37°C overnight. After centrifugation, the supernatant was mixed with 400 μL of 3 M sodium acetate and 10 mL of binding buffer (5 M GuHCl, 40% isopropanol, 0.05% Tween‐20) and transferred into silica columns. Washing was performed with 450 μL of PE buffer and DNA eluted with 45 μL of EBT buffer (10 mM Tris–HCl pH 8.5, 0.05% Tween‐20). Two extraction blanks, containing reagents alone and molecular grade water instead of calculus or soil samples, positioned as the first and last tubes, were included in every extraction batch of up to 16 samples to control for laboratory and reagent contamination.

Following DNA extraction, double‐stranded genomic libraries (Meyer and Kircher 2010) were constructed using 20 μL of the DNA. We used the double‐barcoding double‐indexing strategy (Van Der Valk et al. 2020) to identify and later remove instances of index hopping. One library blank, containing molecular grade water instead of DNA, was included in every batch of up to 16 samples. The appropriate number of indexing cycles was estimated using a quantitative PCR assay and ranged from 12 to 20 (Table S1). Qiagen MinElute columns were then used to clean up the libraries, and size selection was performed with Agencourt AMPure XP PCR Purification beads (Beckman Coulter, IN, USA) to remove adapter dimers and fragments above 500 bp. The libraries were pooled (using 1.5 μL of each negative control and 3 μL of each dental calculus and soil sample) and submitted to SciLifeLab (Uppsala, Sweden) for shotgun sequencing on two lanes of an S4 flow cell using the NovaSeq 6000 system and v1.5 sequencing chemistry (150 bp PE).

### 2.4 Data Processing

All scripts, specifying flags and options, are available on https://github.com/markella‐moraitou/Roman_oral_microbiomes. After demultiplexing, we processed the sequences using the nf‐core/eager pipeline (Fellows Yates, Lamnidis, et al. 2021) (v. 2.4.4, available at https://nf‐co.re/eager/2.4.4), which performs read merging and adapter removal, filtering, mapping to the human genome and taxonomic classification of unmapped reads using Kraken2. We kept the default parameters, except for the minimum read length in the adapter clipping and read merging step (adjusted to 35 bp) and the minimum base quality (30). Unmerged reads were excluded from downstream analysis. For the BAM filtering step, the minimum mapping quality for reads and the minimum read length to be kept after mapping were set to 30 and 35 bp, respectively. Mapping against the human reference genome (GRCh38) was performed in the nf‐core/eager pipeline using BWA‐aln (Li and Durbin 2009) and unmapped reads were converted to FASTQ format and used downstream for the microbial and dietary analyses. Source tracking was performed using decOM (v. 0.0.32) (Duitama González et al. 2023), a k‐mer‐based method developed specifically for dental calculus metagenomes. We used the default source matrix, which is made up of ancient and modern human oral microbiomes, sediment/soil and skin.

### 2.5 Taxonomic Analysis

Taxonomic classification was carried out using the standard database of Kraken2 (v. 2.1.6) (Wood et al. 2019), which includes all the bacteria, archaea and virus genomes available on NCBI. Bracken (v. 2.6.2) (Lu et al. 2017) was used to estimate abundances at the species level, and the *taxize* R package (v. 0.9.100) (Chamberlain and Szöcs 2013) was used to retrieve taxonomic information. Microbial taxon abundances (Table S2), taxonomic information and sample metadata were analyzed jointly using the *phyloseq* R package (v. 1.48.0) (McMurdie and Holmes 2013).

We only retained dental calculus samples with an oral content of at least 75% according to decOM (ancient and modern oral partition combined). To evaluate whether the ‘Unknown’ source fraction, which is a decOM feature under development, influenced which samples are removed, we also calculated a metric that is independent of it: the ratio of oral content to likely contaminants (skin and sediment/soil). We then performed abundance filtering by converting any relative abundances accounting for less than 0.01% for a given sample to zero. As some of the microbial taxa found in dental calculus are likely contaminants from the burial environment, we used information from co‐extracted soil samples to identify them. Specifically, taxa with a higher relative abundance in any single soil sample than in any dental calculus sample were considered to be derived from the soil microbiome and removed from dental calculus samples and blanks as environmental contaminants.

To assess alpha diversity, we produced rarefaction curves (with subsample size ranging from 100 to 10,000 reads) using the ‘plot_alpha_rcurve’ function from the *microbiomeutilities* R package (v. 1.00.17) (Shetty and Lahti 2020). We analyzed alpha diversity only for samples from healthy teeth that had reached or were near the species richness asymptote. Specifically, we tested for the effect of cemetery provenance on the observed species richness and the Shannon evenness index using Type II (hierarchical) ANOVA (Analysis of Variance). Although only healthy teeth were analyzed, some included individuals had caries elsewhere in the oral cavity. Therefore, individual health state and sample read count (after abundance re‐estimation with Bracken) were included as explanatory variables to account for potential effects. We used Shapiro–Wilk tests to assess whether the ANOVA residuals meet the normality assumption and tests were interpreted when the deviation from normality was not significant (*p* > 0.05), or significant but small (*W* > 0.95).

To investigate the composition of the oral microbiome, we conducted all analyses with taxonomic assignments at the species level and applied a centered‐log‐ratio (CLR) normalization using the *microbiome* R package (v. 1.26.0) (Lahti and Shetty 2018). We used Aitchison distances (Aitchison 1982), which are defined as the Euclidean distances between CLR‐transformed abundances to perform permutational analysis of variance (PERMANOVA) and assess the marginal effects of sample read count, cemetery and health state, using the *vegan* R package (v. 2.6–8) (Oksanen et al. 2011). As our dataset includes pairs of samples from caries and healthy teeth from the same individuals, to avoid violating the assumption of independence, we included only one sample per individual. We therefore ran two PERMANOVA tests: One including only healthy teeth, so that the health status factor reflected the oral health of the individual, and another replacing healthy teeth with carious ones where both healthy and diseased teeth were available for the same individual, so that the health status reflected tooth health. For any significant multi‐level factor, we performed pairwise PERMANOVAs using the “pairwise.adonis” function from the *pairwiseAdonis* R package (v. 0.4.1) (Martinez Arbizu 2020). We identified differentially abundant taxa while accounting for unequal sequencing depth across metagenomes with the *ANCOMBC2* R package (v. 2.6.0) (Lin et al. 2022; Lin and Peddada 2020), limiting the test to taxa present in at least 10% of samples and with at least 1000 reads. We adjusted the *p* values for multiple testing using the Benjamini‐Hochberg method. Principal coordinate analysis (PCoA) plots were generated from CLR‐transformed data with the *phyloseq* and *ggplot2* R packages (v. 3.5.1) (Wickham 2016).

To gain further insight into the health status of these communities, we assessed the presence and abundance of oral pathogens associated with caries and periodontitis, as reported in HOMD (Chen et al. 2010) and Zhang et al. (2022). Species names were in some cases amended to match NCBI taxonomy. In addition, to evaluate potentially systemic impacts of the oral microbiome, we looked into taxa implicated in endocarditis (including the HACEK group; Cahill and Prendergast 2016), as this condition is often caused by ectopic oral microorganisms. We also compared the abundances of early (*Streptococcus*, *Actinomyces*) and late plaque colonizers (red complex bacteria, *Methonobrevibacter*) to evaluate the maturation stage of the dental calculus biofilm. The differences in the ratio of late to early colonizers were tested using ANOVA, as described above for species richness.

### 2.6 Authentication

To confirm that identified taxa were ancient and not the result of modern contamination, we assessed the presence of typical post‐mortem DNA damage patterns for the 20 most abundant taxa (on average) in each cemetery, as well as any differentially abundant taxa, any oral taxa (according to the Human Oral Microbiome Database; HOMD) found in laboratory blanks, and any detected putative pathogens. To do this, we selected the sample with the highest abundance of the focal taxon and mapped the reads to the publicly available reference genome of that taxon (retrieved from NCBI, Table S3) using BWA‐aln (v. 0.7.19‐r1273; flags: ‐n 0.04 ‐k 2 ‐l 1024 ‐o 2). We then converted the alignment to BAM format, extracted mapped reads using SAMtools (v. 1.22.1) and visualized the damage patterns using mapDamage (v. 2.2.3) (Jónsson et al. 2013). We could not perform this analysis for *Streptomyces* sp. M56, as no genome was available for this taxon.

### 2.7 Functional Analysis

Functional analysis was carried out using HUMANn3 (v. 3.9) (Beghini et al. 2021). We used the relative abundance of gene families normalized to copies per million, which were aggregated into GO terms to obtain the functional profiles of the metagenomes (Ashburner et al. 2000; The Gene Ontology Consortium et al. 2021). We only retained GO terms reflecting biological processes (“BP”) and normalized abundances using a CLR transformation. We then visualized the functional composition of soil and dental calculus microbiomes using PCoA ordinations implemented in the *phyloseq* R package. Dental calculus samples clustering with soil were considered poorly preserved and removed. The retained dental calculus samples were used to test for differences in the functional capacity among the oral microbiomes of the three populations, using PERMANOVA with Aitchison distances and controlling for sequencing depth (number of reads per sample). For any significant factors, we performed between‐level comparisons using the “pairwise.adonis” function from *pairwiseAdonis*, and differential analysis using *ANCOMBC2* (this time using a prevalence cutoff of 20%).

### 2.8 Sex Assignment

Molecular sex identification was performed using sexassign (commit 94b4783) (Gower et al. 2019). Following mapping to the human reference genome as part of eager, we filtered the resulting BAM files (mapping quality ≥ 30, length ≥ 35 bp) and removed reads that mapped to unplaced scaffolds (using samtools view to extract each chromosome and samtools merge to combine them) (Li et al. 2009). We generated coverage statistics using samtools idxstats (Li et al. 2009) as input for sexassign, which uses a probabilistic approach based on the ratio of reads mapping to the X chromosome versus the autosomes to identify the sex of the sampled individuals. A likelihood ratio near 0.5 corresponds to males, a ratio near 1.0 corresponds to females, while sex cannot be assigned for ratios between 0.6 and 0.8 (Gower et al. 2019).

### 2.9 Inferring Likely Geographic Origin of the Study Individuals

To obtain insights into the genetic affinities of the study individuals, we first constructed a BWA index for the human reference genome hg19. Next, we aligned the sequence reads to this reference using BWA‐aln with settings optimized for ancient DNA (‐l 16,500 ‐n 0.01 ‐o 2) and randomly sampled an allele at each site where reads mapped using ANGSD (‐dohaplocall 1 ‐doCounts 1 ‐remove_bads 1 ‐uniqueOnly 1 ‐minMapQ 20) (Korneliussen et al. 2014). We then used 2506 human genomes available in the 1000 Genomes Project Phase 3 (Sudmant et al. 2015; The 1000 Genomes Project Consortium et al. 2015) and identified variable positions in this reference dataset that were also covered by at least 1 read in our data. To identify the genetically closest reference population for each study individual, we counted the number of matching alleles between our samples and each of the 2506 human reference genomes. The population with the fewest mismatches per variable site was considered to be the closest present‐day population to the ancient samples.

### 2.10 Dietary Analysis

To minimize the misclassification of microbial reads as plant or mammalian reads of interest, sequencing reads were first classified against the Genome Taxonomy Database (GTDB) v220 (Parks et al. 2022). Reads that remained unclassified were subsequently classified against the PlusPFP database, which comprises RefSeq archaeal, bacterial, viral, plasmid, human, UniVec_Core, protozoan, fungal and plant sequences (database retrieved on 28 December 2024) (O'Leary et al. 2016). The reads still unclassified after these two steps were retained for downstream analyses, as they were considered depleted of most microbial and common contaminant sequences. Although some plant reads may have been removed during the second classification step, the impact on plant‐derived reads is expected to be minimal given the limited representation of plant reference genomes in the database.

To obtain dietary insights from the dental calculus samples, we classified these retained sequencing reads to plants and animals. First, we constructed genome‐wide databases for plants and mammals using Kraken2 (v 2.1.3) (Table S4). The Kraken2 plant database was built by sampling 1257 NCBI genome accessions of different plant species from different genera, all under the family *Viridiplantae*, all Refseq mitochondrial genomes available in April 2023 and all assemblies available in the PhyloNorway database, which contains skimmed genomes of 1323 species (retrieved 2023.4.13). In total, this database contained 2638 plant species from 1087 genera, of which PhyloNorway and NCBI assemblies together contribute 2376 species from 960 genera (Jin et al. 2025). A second Kraken2 database for mammals was built using 740 genomes of mammalian species belonging to 412 genera. We classified all merged reads against both these databases in Kraken2 with default settings, using the ‐‐report‐minimizer‐data parameters to obtain the number of unique kmers assigned to each species. Additionally, since classical literature and modern studies point to the consumption of marine resources by these populations, we built a bowtie2 index for 27 fish genomes (Table S4) that could have been consumed at the time (Craig et al. 2009; Locker 2007; Marzano 2013, 2018; Purcell 1995). We then competitively mapped all the merged reads against this single bowtie index using bowtie2, with the –very‐sensitive setting, and subsequently used samtools depth to count the number of bases covered for each of the fish genomes.

Reliable identification of eukaryotic sequences, particularly of dietary nature, from dental calculus is challenging (Fagernäs et al. 2022; Mann et al. 2023). We, therefore, implemented several pre‐processing steps to reduce the detection of false positives. Specifically, we only retained eukaryotic taxa with at least 100 reads (or at least 100 mapped sites for fish) in dental calculus samples, and used soil samples as environmental controls, excluding taxa as likely environmental contaminants if the average relative abundance in soil samples (or negative laboratory controls) was higher than the average relative abundance in dental calculus samples. Since Isola Sacra lacked soil samples and only a single soil sample was available from Selvicciola, we combined the information from all available soil samples for this analysis.

## 3 Results

### 3.1 Data Processing, Validation and Alpha Diversity

We collected 71 dental calculus and eight soil samples from three cemeteries in Central Italy (Table 1). Our dataset also included 16 negative controls (including extraction and library preparation negatives) that were processed alongside the samples (Table S1).

We obtained a mean of 65,472,308 paired reads per dental calculus sample (ranging from 4,141,846 to 144,842,179), a mean of 43,495,561 paired reads per soil sample (6,448,503–99,467,428), and a mean of 171,139 paired reads from the negative controls (654–1,160,363) (Table S1). Thus, negative controls produced at least an order of magnitude fewer reads than samples.

**FIGURE 2:**
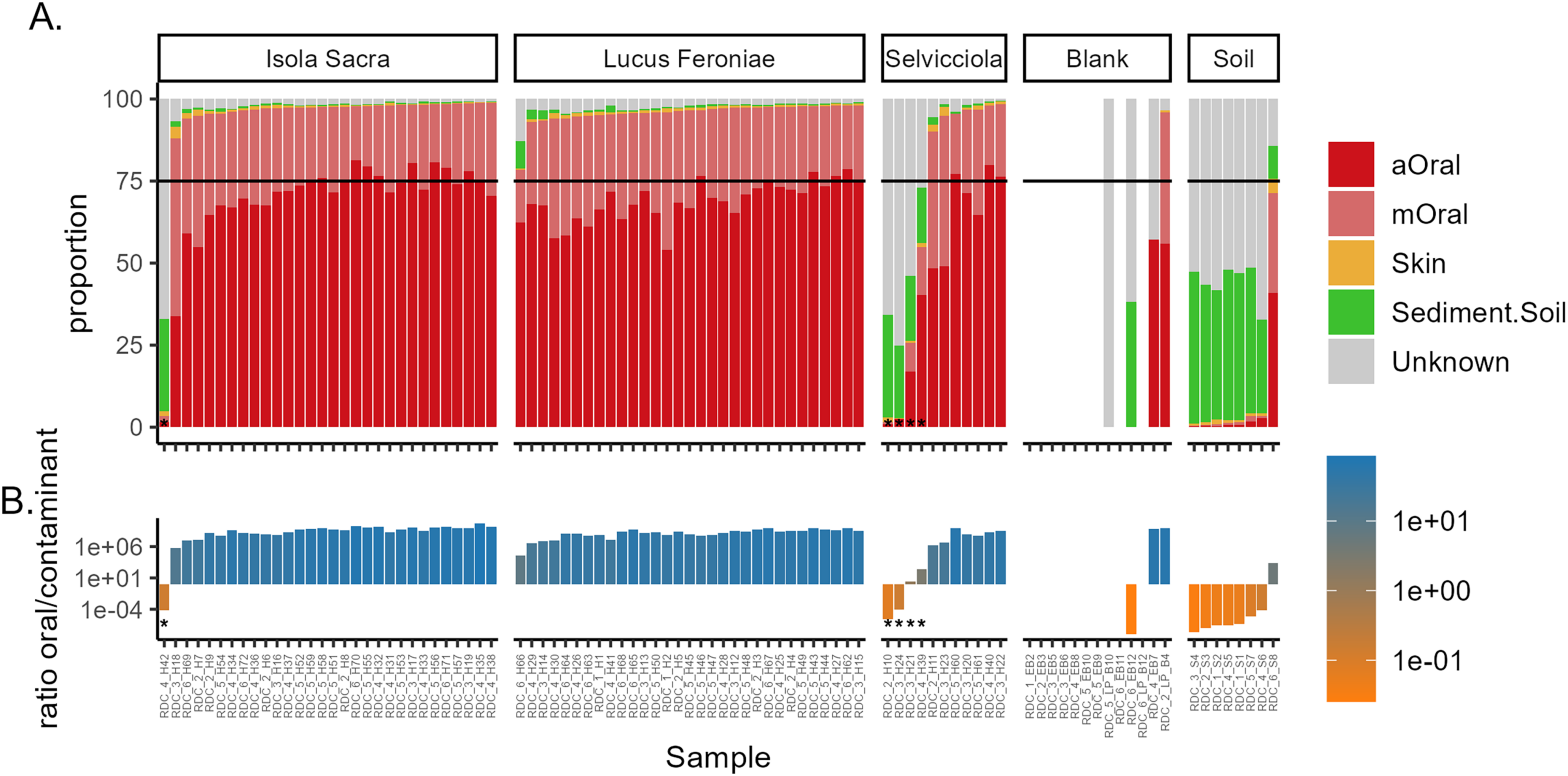
DecOM source tracking output. (A) Estimated proportion per sample resembling ancient (aOral) and modern (mOral) oral metagenomes, skin and sediment/soil. The “Unknown” category reflects fractions of the metagenomes that did not match any of the sources. Bars in white indicate metagenomes with insufficient information to generate an output (in all cases from blanks). Black line at 75% denotes the cut‐off for human oral microbiome proportion, below which samples were deemed to be poorly preserved and removed from further analyses. (B) Ratio of oral to contaminant (sum of skin and soil/sediment) proportions for the samples in Panel A (note log‐transformed *y*‐axis). Pseudo‐counts of 1% were added to both proportions. Samples with asterisks were excluded due to low preservation (see also Table S1).

We taxonomically classified the samples using the standard Kraken2 database (Wood et al. 2019) and identified a total of 1953 microbial species (1902 of these found in dental calculus, 1506 in soil and 107 in blanks). Source tracking using decOM suggested that, on average, 90.8% of dental calculus metagenome composition resembled the human oral microbiome (ancient and oral fractions combined; range 1.5%–98.9%), whereas this fraction was 11.8% for blanks (range: 0%–96.0%) and 10.4% for soil (range 0.42%–71.46%, Figure 2A). Dental calculus samples with less than 75% oral proportion were considered to be poorly preserved and, accordingly, five such samples were excluded from downstream analysis (Table S1). As the “Unknown” source proportion estimated by decOM is a feature under development, we also calculated an independent metric: the ratio of the oral proportion (ancient and modern) to the exogenous proportion (skin and soil/sediment), which validated our filtering strategy, as it showed that all excluded samples had lower values than the retained ones (Figure 2B).

By comparing the relative abundances of taxa in dental calculus and in soil, we removed 450 likely environmental contaminants. These included 161 known laboratory contaminants (Salter et al. 2014; Weyrich et al. 2019), 12 of which are also oral taxa ( *Staphylococcus epidermidis* , *Staph. pettenkoferi*, *Staph. pseudoxylosus*, *Streptococcus pyogenes* , *Actinomyces oris* and seven *Corynebacterium* species) (Chen et al. 2010; Fellows Yates, Velsko, et al. 2021). After removing these taxa from dental calculus and blank samples, and performing abundance filtering with a 0.01% relative abundance threshold, we retained 1369 taxa in the complete dataset (919 of these found in dental calculus, 1025 in soil and 68 in blanks; Table S5). Following this decontamination procedure, dental calculus, soil and blanks formed separate clusters in a PCoA ordination using Aitchison distances (Figure S1). Of the 68 taxa in blanks, nine were oral taxa present in the HOMD: *Staphylococcus cohnii* , *Staph. hominis*, *Streptococcus gordonii* , *Lactococcus lactis* , *Methanobrevibacter* sp. YE315, *M. smithii* , *Corynebacterium hindlerae*, *Prevotella* sp. oral taxon 299 and *Neisseria meningitidis* . These oral taxa tended to be among the most abundant in samples from the same extraction or library preparation batches, or across the dataset (Figure S2), suggesting that they reflect cross‐contamination from dental calculus during laboratory procedures rather than external contamination. Further supporting this, all of them showed aDNA‐associated damage patterns (Figure S3).

Rarefaction curves suggested that the number of identified microbial taxa in a dental calculus sample reaches an asymptote after roughly 4000 taxonomically classified reads (after filtering and decontamination) (Figure S4). For this reason, samples with fewer than 4000 reads were not considered in ANOVA tests assessing microbiome species richness and evenness (Figure S5, Table 2, and Table S1). Both richness and evenness tests met the normality of the residuals assumption (Shapiro–Wilk: *W* = 0.96, *p* value = 0.1 for richness; *W* = 0.98, *p* value = 0.38 for evenness). We did not detect any differences in species richness or evenness by cemetery, although read count affected evenness, even after excluding samples with too few reads (Table 2).

**TABLE 2:** Species richness and evenness of the dental calculus microbiome of healthy teeth do not differ across cemeteries after accounting for read count and health state (i.e., presence of caries elsewhere in the oral cavity).

Response	Observed species richness		Shannon index	
	*F*‐statistic	*p*	*F*‐statistic	*p*
Read count	1.945	0.170	19.589	**< 0.001**
Individual health state	1.805	0.186	2.262	0.140
Cemetery	1.826	0.172	1.971	0.150

*Note:* Results of ANOVA tests with Type II sums of squares for observed species richness and the Shannon index. This analysis included 52 samples from healthy teeth with at least 4000 classified reads (23 from Isola Sacra, 24 from Lucus Feroniae, 5 from Selvicciola). Statistically significant results (*p* value < 0.05) are highlighted in bold.

### 3.2 Oral Microbiome Composition and Function Are Associated With Differences in Lifestyle

**FIGURE 3:**
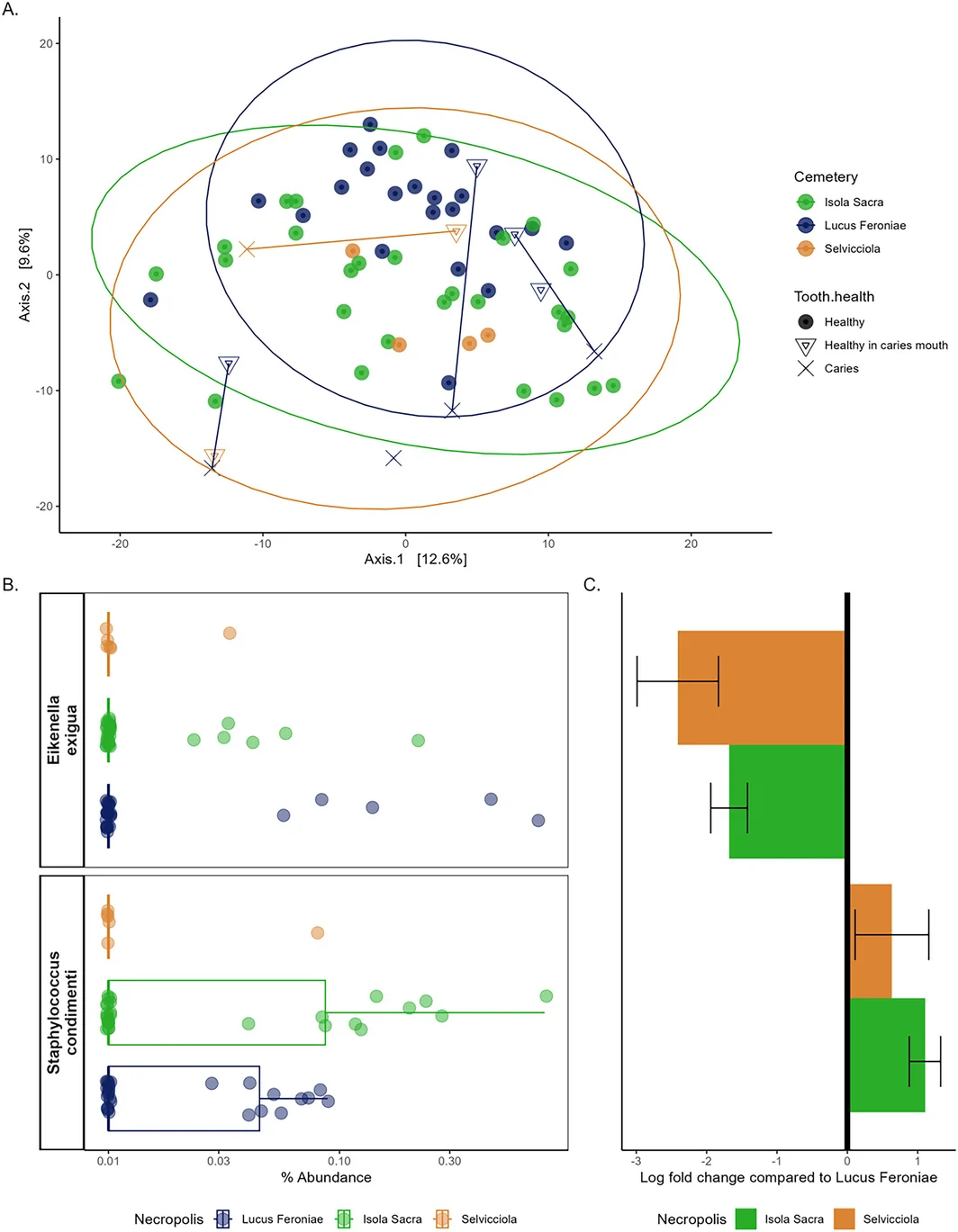
(A) Compositional differences between 66 dental calculus samples that were retained after filtering and decontamination: 29 from Isola Sacra (in green), 30 from Lucus Feroniae (in blue) and seven from Selvicciola (in orange). Five caries samples (cross symbols) and six samples of healthy teeth in individuals with caries (triangle symbols) are included. Four pairs of healthy‐caries teeth from the same individual are connected by a line. The ellipses show the 95% confidence level using a multivariate *t*‐distribution. (B) Relative abundances of differentially abundant microbial species between Lucus Feroniae and Isola Sacra, identified using *ANCOMBC2* with Lucus Feroniae used as the reference. None of the comparisons passed the *ANCOMBC2* sensitivity analysis. Note the log‐transformed (with 1% pseudo‐count) *x*‐axis. (C) Log‐fold taxon abundance changes compared to Lucus Feroniae for the microbial species in B, as estimated by *ANCOMBC2*. The error bars reflect the standard error of the log‐fold change.

To investigate whether the differences in social status and time period that characterize the three cemeteries affected the oral microbiome, we compared microbial compositions across the cemeteries using PERMANOVA with Aitchison distances on samples from healthy teeth (*N* = 61, excluding the five caries samples). We estimated the marginal effects of sequencing depth, individual health state and cemetery provenance, and observed only a small but statistically significant effect of cemetery provenance on the dental calculus bacterial communities (Table 3 and Figure 3A). Specifically, we found that the Classical period cemetery of Lucus Feroniae differed significantly from both the contemporaneous cemetery of Isola Sacra and the post‐Classical cemetery Selvicciola (Table 3 and Figure 3A).

**TABLE 3:** PERMANOVA and pairwise PERMANOVA using Aitchison distances on taxon abundances, presenting the effect size (*R* ^2^) and *p* value of read count (as a control for sequencing depth), health state, and cemetery provenance.

Using samples from healthy teeth only, including six healthy samples from individuals who had caries elsewhere in the mouth			
PERMANOVA terms	SumOfSqs	*R* ^2^	*p*
Read count	615	0.020	0.222
Individual health state	529	0.015	0.407
Cemetery	1465	0.045	**0.036**
Residuals	29,622	0.915	

Pairwise PERMANOVA	SumOfSqs	*R* ^2^	Adjusted *p* value (Benjamini‐Hochberg)
Isola Sacra vs. Lucus Feroniae	884.89	0.031	**0.033**
Isola Sacra vs. Selvicciola	587.51	0.028	0.443
Lucus Feroniae vs. Selvicciola	804.82	0.054	**0.033**

Using healthy teeth from individuals without caries and replacing healthy teeth with caries samples for five individuals who had both sample types			
PERMANOVA terms	SumOfSqs	*R* ^2^	*p*
Read count	665	0.020	0.194
Tooth health	1177	0.036	**0.003**
Cemetery	1211	0.037	0.234
Residuals	29,870	0.908	

*Note:* To avoid including multiple samples from the same individual, the first PERMANOVA contained only samples from healthy teeth (*N* = 61, 29 from Isola Sacra, 26 from Lucus Feroniae, and 6 from Selvicciola) and tested for the individual's health state. The second PERMANOVA included the carious teeth from those individuals that had both healthy and diseased teeth available, and tested for the health status of the tooth (caries or healthy) (*N* = 60 samples, 5 samples for Selvicciola). Statistically significant results (*p* value < 0.05) are highlighted in bold.

However, when comparing carious teeth with samples from individuals without caries, we found that tooth health was the only significant predictor, obscuring cemetery provenance (Table 3). Nevertheless, carious teeth did not cluster together and instead appeared more dispersed compared to their healthy counterparts, occupying the margins of the PCoA plot, a pattern also observed in an additional caries sample from Lucus Feroniae without a healthy pair (Figure 3A). Although the sample size was too small to draw general conclusions, this could potentially reflect the variable microbiome composition of caries microbial communities.

Given the stark differences in microbiome composition between healthy and diseased teeth, we screened for a catalogue of putative pathogens of oral origin (Table S6). Considering the entire dataset, we detected four caries‐associated taxa (but not the canonical *Streptococcus mutans* , which was absent), as well as 12 taxa associated with endocarditis, a heart infection often caused by ectopic oral bacteria (Figure S6). One of the most abundant taxa, *Streptococcus gordonii* (Table S5), is a putative heart pathogen but also a normal part of the oral microflora (Mosailova et al. 2019). This holds for all endocarditis‐associated taxa: they do not cause the disease unless they enter the bloodstream (Cahill and Prendergast 2016). Periodontal pathogens, including the red complex bacteria *Porphyromonas gingivalis* , *Tannerella forsythia* , and *Treponema denticola* (Socransky et al. 1998), were absent from the dataset (Table S6). All of the detected pathogens (Figure S6) exhibited aDNA‐associated damage patterns, except for *Kingella oralis* whose low number of reads was insufficient for damage pattern analysis (Figure S3). Surprisingly, when considering the four caries‐associated taxa detected in the dataset, only *Ligolactobacillus salivarius* was present in any caries sample (Figure S6). The remaining three taxa, *Leptotrichia shahii* , *Prevotella melaninogenica* , and *Bifidobacterium longum* , were present in healthy teeth only (Figure S6).

Differential abundance analysis was performed with *ANCOMBC2* and, since Lucus Feroniae differed from both Isola Sacra and Selvicciola, it was used as the reference group. Despite the differences in microbiome composition between Lucus Feroniae and Selvicciola (Table 3 and Figure 3A), we were unable to identify any differentially abundant microbial species between the two cemeteries, likely due to the low sample size of Selvicciola (*N* = 6 retained samples). However, we identified two taxa that differ in abundance between the contemporaneous cemeteries of Lucus Feroniae and Isola Sacra: *Eikenella exigua* was enriched in Lucus Feroniae, and *Staphylococcus condimenti* was enriched in Isola Sacra (ANCOMBC2 adjusted *p* value < 0.05; Figure 3B,C). Selvicciola showed the same trends as Isola Sacra (Figure 3C). However, neither taxon passed the *ANCOMBC2* sensitivity analysis, suggesting they were inconsistently identified depending on the pseudo‐count used by the software. Neither are part of the HOMD (Chen et al. 2010), but other species belonging to the same genera are, and the presence of damage patterns suggests these are authentic ancient taxa (Figure S3). None of the caries‐ or endocarditis‐associated taxa reported above (Figure S6) differed in abundance across cemeteries after adjusting for multiple testing. However, the putative heart pathogens *S. gordonii* and *S. salivarius* appear depleted in Isola Sacra before adjusting (*ANCOMBC2 p* value < 0.05), with a similar nonsignificant trend in Selvicciola.

To evaluate differences in dental calculus maturation stage in our dataset, we compared the total abundance of early colonizers of dental plaque (*Streptococcus* and *Actinomyces*) to that of late colonizers (specifically *Methanobrevibacter*, as the red complex bacteria were absent). Most samples had a higher proportion of early colonizers, with 10 exceptions, seven of which were samples from Isola Sacra (Figure S7). Despite this, we found that neither cemetery nor health status (individual with or without caries) explained the abundance ratio between *Methanobrevibacter* and early colonizers (ANOVA *p* value > 0.17; Shapiro–Wilk: *W* = 0.95, *p* value = 0.02), with read count being the only significant predictor (*p* value = 0.02). Information on sex and age, which might be related to increase in *Methanobrevibacter*, was not available for these samples (Table S1).

**FIGURE 4:**
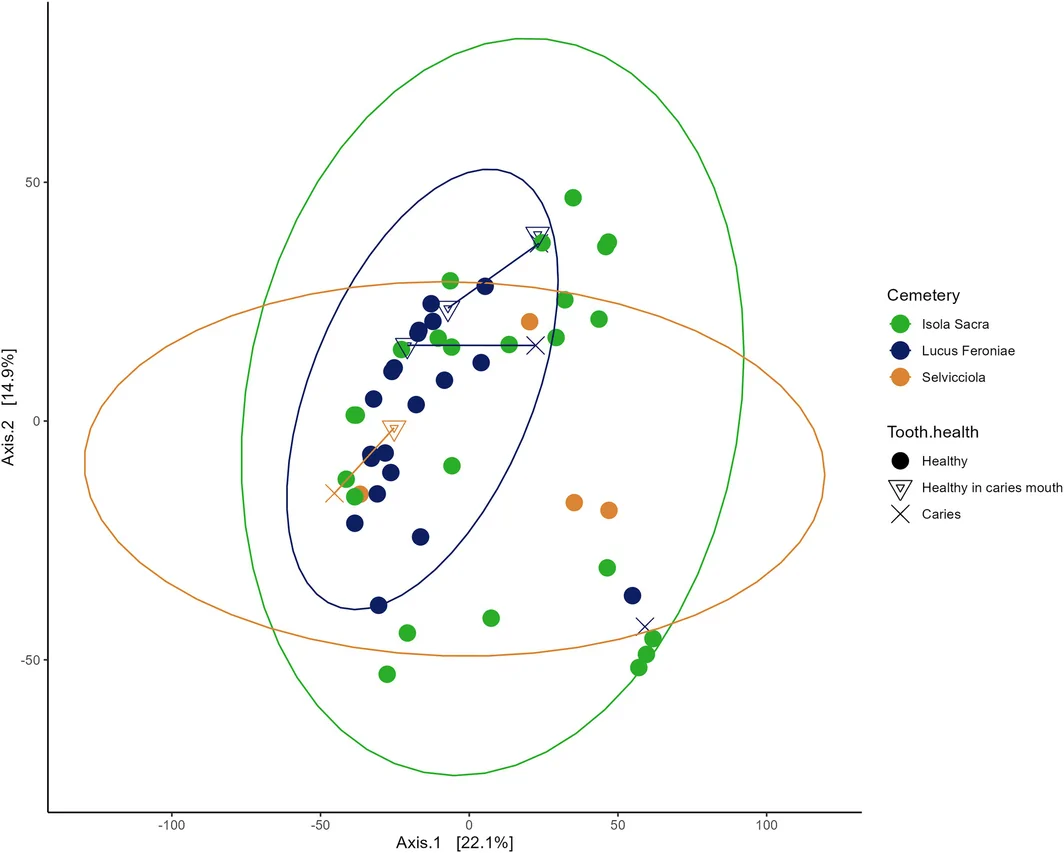
Functional profiles of the 56 retained dental calculus samples from Isola Sacra (in green), Lucus Feroniae (in blue) and Selvicciola (in orange) using CLR‐transformed relative abundances of GO terms referring to biological processes. Four caries samples (cross symbols) and four samples of healthy teeth in individuals with caries (triangle symbols) are included. Three pairs of healthy‐caries teeth from the same individual are connected by a line. The ellipses show the 95% confidence level using a multivariate *t*‐distribution.

To understand whether the three populations differed in the functional capacity (in terms of gene content) of their oral microbiomes, we compared the compositions of Gene Ontology (GO) terms referring to biological processes. We visualized these functional compositions for both soil and dental calculus microbiomes using PCoA and identified 10 dental calculus samples that clustered with soil, indicating poor preservation (Figure S8). As GO terms are often encoded by multiple microbial taxa, decontamination based on taxon abundance, as performed for taxonomic analyses, was not possible. Instead, we removed these 10 samples from downstream analysis, retaining 56 dental calculus samples (24 from Isola Sacra, 26 from Lucus Feroniae, 6 from Selvicciola) and 1100 GO terms. After accounting for differences in sequencing depth, PERMANOVA (without caries samples) showed a significant difference in microbial functions across cemeteries (Figure 4, Table 4, and Table S7). As was the case for the taxonomic characterization, Lucus Feroniae was significantly different from the other two cemeteries (Table 4).

**TABLE 4:** PERMANOVA results for differences in functional potential across the cemeteries, for 52 dental calculus samples after excluding poorly preserved and caries samples, showing the effect size (*R* ^2^) and *p* value for read count (as a control for sequencing depth), health state (presence of caries in the sampled individual), and cemetery provenance for the dental calculus samples from Isola Sacra, Lucus Feroniae, and Selvicciola based on Aitchison distance.

PERMANOVA terms	SumOfSqs	*R* ^2^	*p* value
Read count	16,137	0.068	**0.001**
Cemetery	15,817	0.066	**0.018**
Individual health state	3599	0.015	0.596
Residuals	201,684	0.846	

Pairwise PERMANOVA	SumOfSqs	*R* ^2^	Adjusted *p* value (Benjamini‐Hochberg)
Isola Sacra vs. Lucus Feroniae	12128.632	0.057	**0.015**
Isola Sacra vs. Selvicciola	4231.863	0.027	0.668
Lucus Feroniae vs. Selvicciola	6947.173	0.069	**0.037**

*Note:* Statistically significant results (*p* value < 0.05) are highlighted in bold.

Only one biological process was significantly enriched in Isola Sacra compared to Lucus Feroniae: GO:0061077, chaperone‐mediated protein folding, which is mostly encoded by *Neisseria* and *Eikenella*. It also appeared enriched in Selvicciola; however, the comparison was not significant, possibly due to the small number of samples retained from this cemetery (*N* = 6).

### 3.3 Dietary Analysis

After removing eukaryotic taxa with higher relative abundance in soil and control samples than in dental calculus samples (Table S8), we detected 30 plant genera, 23 mammalian genera, and 17 fish species in the decontaminated and filtered dataset (Table S9). The implemented decontamination procedure allowed us to remove several contaminants and spurious hits, including plant species introduced into Europe from the Americas long after the study period and, hence, representing obvious false positives. However, despite these steps, one New World genus (*Sequoiadendron*) remained in the decontaminated plant dataset, and 10 New World genera were present in the mammalian dataset, suggesting the retention of contaminant or spurious taxa (Table S8).

Each retained plant taxon showed, on average, 163.18 reads per sample (171.75 in dental calculus, 100.26 in soil and 1.39 in blanks, Table S10). All 30 plant taxa were detected in all three cemeteries. Five of them are plausible dietary taxa (*Brassica*, *Cornus*, *Prunus*, *Triticum* and *Vitis*), which were likely consumed by the individuals under study and still represent important food sources today (except *Cornus*). However, as these taxa were also detected in the co‐analyzed soil samples, suggesting their presence in the environment, direct consumption remains uncertain. The other detected genera were most likely not of dietary relevance but rather occurred in the environments surrounding the sites and cemeteries under study (e.g., *Juncus*, *Rhynchospora*), as they still occur in Italy today. Some taxa may have been used for timber (e.g., *Ilex*, *Populus*, *Quercus*) or for their medicinal properties (e.g., *Cardamine*, *Chenopodium*, *Trifolium*).

Each retained mammalian taxon presented, on average, 176.34 reads per sample (188.05 in dental calculus, 85.88 in soil and 1.08 in blanks). However, only 13 genera were likely present during Roman times, as the others are native to the Americas. Only two mammalian genera may have had dietary relevance in Italy (*Equus* and *Lepus*). As for the plant taxa, the mammalian genera detected could be naturally occurring in the environments around the archaeological sites, as they are also present in the soil samples.

Each retained fish taxon showed an average of 379.75 mapped sites per sample (427.18 in dental calculus, 704.92 in soil and 3.75 in blanks). All the species recovered, or their respective genera, may have been consumed as food, with the only exception of *Gymnothorax javanicus* , due to its potential toxicity (Dao et al. 2025), and *Doryteuthis pealeii*, likely not present in the region, as its native range is in the northwestern to central Atlantic. The species *Dicentrarchus labrax* , *Merluccius merluccius* , *Mullus surmuletus* , *Mytilus galloprovincialis* , and *Sparus aurata* are present in the Mediterranean Sea and have been reported in the literature as having been consumed during Roman times (Marzano 2013).

### 3.4 Sex Assignment

Of the 67 study individuals, 14 had ≥ 1000 reads mapping to the X chromosome and the autosomes, and we therefore attempted molecular sex identification. Eleven samples were from dental calculus, whereas the remaining three samples were from skeleton‐associated soils. These samples were collected by dislodging soil particles attached to human bones (Table S1) and are, therefore, likely to contain human DNA that leaked into the sediments during the decomposition period (Emmons et al. 2017). None of the soil samples had a paired dental calculus sample with a sufficient number of reads for molecular sex assignment.

We were able to assign molecular sex to seven individuals based on dental calculus samples and to all three soil‐based samples (Table 5 and Table S11). Sex assignment based on morphological features was available for eight individuals (Table 5) (Argenti and Manzi 1988; Micarelli 2020; Tafuri et al. 2018), allowing comparisons between molecular and morphological sex for six individuals. In four cases, morphological and molecular data were in agreement, whereas in two cases (one from dental calculus and one from soil) they disagreed (Table 5) (Argenti and Manzi 1988; Tafuri et al. 2018). However, we note that the molecular sex assignment to individual RDC_2_H10 was less robust, with the likelihood ratio of X versus autosomes close to 0.8 (Table S11). The discrepancies between genetic and skeletal sex can be due to various factors, including the state of conservation of the material, contamination, and insufficient number of human reads.

**TABLE 5:** Skeletal and genetic sex assignments for 11 dental calculus samples and 3 soil samples.

Cemetery	Source	Lab ID	Museum ID	Skeletal sex	Molecular sex
Lucus Feroniae	Dental calculus	RDC_2_H3	LFR Prato della Corte Polimeni T11	F	ND
**Selvicciola**	**Dental calculus**	**RDC_2_H10**	**SLV 86/1**	**M**	**F?**
Selvicciola	Dental calculus	RDC_2_H11	SLV 85/6	M	M
Lucus Feroniae	Dental calculus	RDC_3_H14	LFR PLN T4 E4	ND	F
Isola Sacra	Dental calculus	RDC_3_H16	NIS 45 F1B.D	ND	M
Selvicciola	Dental calculus	RDC_3_H23	SLV T89/5 B	M	ND
Selvicciola	Dental calculus	RDC_4_H39	SLV T85/7 A	M	M
Lucus Feroniae	Dental calculus	RDC_5_H44	LFR MRT D41 A	ND	F
Selvicciola	Dental calculus	RDC_5_H60	SLV T85/2 xy	M?	M
Lucus Feroniae	Dental calculus	RDC_6_H66	LFR Prato della Corte Polimeni T? B	ND	ND
Isola Sacra	Dental calculus	RDC_6_H69	NIS W. A.	ND	ND
Lucus Feroniae	Soil	RDC_1_S1	LFR Via Tiberina Tomba 7 (vertebra)	F	F
**Lucus Feroniae**	**Soil**	**RDC_4_S5**	**LFR ENEL I A3 (calcaneus)**	**F**	**M**
Lucus Feroniae	Soil	RDC_6_S8	LFR PLN 22 E25 (vertebra)	ND	F

*Note:* Bold entries depict discrepancies in sex identification between the methods.

Abbreviations: F, female; F?, probably female; M, male; M?, probable male; ND, not‐determined.

### 3.5 Geographic Origins of the Study Individuals

Human dental calculus samples typically contain < 1% eukaryotic reads, with the host read proportion being even lower (0.007%–0.47%) (Mann et al. 2018). Nevertheless, we attempted to infer genetic affinities for samples for which at least 10,000 SNPs of the reference panel were covered by at least one sequencing read. Eleven samples satisfied this requirement and consistently showed the closest relationship to Tuscany/Iberia (Table S12). Other samples had too few sites for reliable population ancestry assignments. We are, therefore, unable to confidently infer any movement of individuals buried at the study cemeteries from the human genomic data contained in dental calculus.

## 4 Discussion

Our knowledge of the past is expanding through the combination of bioarchaeological methods applied directly to skeletal remains and the study of historical written records (Martin et al. 2013a, 2013b, 2013c), providing a more holistic view of ancient societies. Here, we used ancient DNA analysis of the microbiome preserved in dental calculus samples to enhance our understanding of the lifestyle of underrepresented social classes from the Classical and post‐Classical periods. As farmers, freemen, slaves, and war veterans are often overlooked in written records (Antonio et al. 2019; Cocozza et al. 2023; Cucina et al. 2006; Redfern 2018; Wright et al. 2024), bioarchaeological methods provide a powerful (and often the only) tool to delve into their lives. In this study, we analyzed dental calculus samples from 67 individuals buried in three cemeteries around the city of Rome (Figure 1): Isola Sacra (I–III century CE), Lucus Feroniae (I–III century CE), and Selvicciola (IV–VIII century CE), exploring not only the taxonomic composition and functional potential of the oral microbiome but also dietary choices and genetic provenance of these individuals.

Our findings suggest that lifestyle, rather than time period, is the most important factor shaping the oral microbiome. Despite the large demographic changes associated with the fall of the Roman Empire, individuals from the Longobard cemetery of Selvicciola do not differ significantly in oral microbiome composition and functional capacity compared to those from the Classical cemetery of Isola Sacra (Tables 3 and 4; Figures 3A and 4). Instead, Lucus Feroniae, which is contemporary with Isola Sacra, differed significantly from the other two cemeteries, both in terms of taxonomic composition and functional potential (Tables 3 and 4; Figures 3A and 4). These observations are consistent with previous evidence that dental calculus microbiomes are generally stable through time (Gancz et al. 2023; Ottoni et al. 2021; Velsko et al. 2023), but are affected by differences in lifestyle (Velsko et al. 2023). Specifically, we know from archaeological evidence and previously published data that Lucus Feroniae differed from the other two cemeteries in the overall health and access to nutrition, which may explain the differences we observe in dental calculus microbiomes.

Individuals buried in the rural town of Lucus Feroniae lived during a flourishing period of the Roman Empire, close to the city of Rome. Their rural inland location likely enabled them to grow food and provided reliable year‐round access to it, as supported by isotopic data (Tafuri et al. 2018). In contrast, individuals buried in Isola Sacra and Selvicciola may have been impacted by a more restricted or unreliable access to food. Although the archaeological record describes the inhabitants of Portus Romae, the town linked to Isola Sacra, as belonging to the middle class (Mannucci and Verduchi 1996), palaeopathological analyses of teeth and long bones suggest sub‐optimal health conditions and a relatively high incidence of physical stressors, possibly related to frequent food crises, as experienced by several Roman cities (Garnsey 1988). Skeletal markers are indicative of poor eating habits during childhood and a more restricted access to resources, particularly when compared to individuals living in Lucus Feroniae (Manzi et al. 1989; Salvadei and Manzi 1997). As an urban coastal town located on the direct commercial route towards the city of Rome, the inhabitants of Isola Sacra likely obtained most of their dietary resources through trade, so the income of food supplies might have been less stable (De Angelis et al. 2020). Individuals buried in Selvicciola, despite being temporally separated and demographically distinct from Isola Sacra, were likely affected by similar nutritional stressors, as revealed by stable isotope analysis showing lower variability in protein sources (Tafuri et al. 2018). Following the collapse of the Roman Empire, the inhabitants of the Post‐Classical site of Selvicciola could no longer rely on the centralized food distribution system (e.g., the *annona* and *frumentatio*) and a stable self‐production system might not yet have been established (Rotili 2010). The low protein and high cereal consumption may explain the high incidence of health problems, both systemic ones, like iron deficiency (indicated by the presence of cribra orbitalia and cribra cranii; Salvadei et al. 2001) and specifically oral ones: dento‐alveolar lesions (Manzi et al. 1999; Salvadei and Manzi 1997), such as caries, abscesses and antemortem tooth loss. Despite this, caries‐associated taxa were not more abundant in Isola Sacra and Selvicciola (Figure S6), suggesting these differences in nutrient availability do not drive the abundances of (putative) pathogens.

While it is remarkable to observe differences in the taxonomic and functional composition of the microbiomes in contemporary and geographically proximate communities, such as Isola Sacra and Lucus Feroniae, these findings highlight the role of potentially similar conditions regarding access to food resources and overall health, before and after the collapse of the Roman Empire. It is, however, worth noting that only 11 dental calculus samples were collected from Selvicciola, four of which were excluded from the taxonomic analysis and an additional two from the functional analysis due to poor preservation (Figures 2 and 4). Hence, our results should be considered with caution.

Despite variations in the oral microbial community composition, we detected only two differentially abundant microbial species across different cemeteries (Figure 3B,C). One such is *Staphylococcus condimenti* , which was more abundant in Isola Sacra (Figure 3B,C) than in Lucus Feroniae. This bacterium is usually found in fish and fermented foods such as soy sauce (Gabrielsen et al. 2017). Its presence could be connected to the consumption of fish, as well as *garum* and *liquamen*, a fermented fish sauce and its byproduct, respectively, commonly used in Ancient Rome and often mentioned in *De Re Coquinaria*, a collection of recipes dated to the I–IV century CE. The higher abundance of *S. condimenti* observed in Isola Sacra could, therefore, be connected to the widespread consumption of fish and fish condiments among the individuals buried there. Being close to the Tyrrhenian coast, people in Isola Sacra might have produced or imported *garum* and *liquamen* to preserve their fish catches, having it more readily available than in Lucus Feroniae. The other taxon, *Eikenella exigua*, was enriched in Isola Sacra and Selvicciola. *Eikenella* is a common genus in dental plaque and may opportunistically act as a pathogen in the oral cavity and other body sites (Chen et al. 1989; Stormo et al. 2020).

In addition to differences across cemeteries, we also observe differences in the microbiome composition, but not alpha diversity, of teeth with caries (Figure 3A, Tables 2 and 3, and Figure S5). The composition of microbial communities from carious teeth is not only different from healthy teeth within the same individual but also tends to occupy peripheral positions in the PCoA space of all considered samples (Figure 3A). While dedicated studies with larger sample sizes are needed to understand the composition of these diseased oral communities in ancient human societies and to compare them with present‐day pathologies, the observed differences are in line with the Anna Karenina principle for dysbiotic microbiomes (Zaneveld et al. 2017). It is also worth noting that caries samples, even those from Lucus Feroniae, we compositionally more similar to microbiomes from Isola Sacra and Selvicciola (Figure 3A). In conjunction with the frequent oral diseases reported in Selvicciola, this may be another indication that the oral microbiome of these two cemeteries reflects an overall poor health state.

The differences and similarities in oral microbiomes that we detect among the study cemeteries are in line with the rich bioarchaeological records, which often suggest nutritional drivers as causes for developmental and health issues (Manzi et al. 1989, 1999; Salvadei and Manzi 1997; Salvadei et al. 2001; Tafuri et al. 2018). However, we were unable to provide direct evidence for dietary profiles from the analyzed dental calculus. We identified the same plant, mammal, and fish taxa in all three cemeteries. As previously demonstrated, extracting dietary information from dental calculus is riddled with challenges (Mann et al. 2023). Plant and animal DNA is considerably less abundant than microbial DNA; eukaryotic databases are not as comprehensive as those used for bacterial and archaea classification; and eukaryotic genomes are usually considerably larger than prokaryotic genomes, increasing the rate of spurious mappings and thereby reducing the information content from the few mapped reads (Mann et al. 2023; Oskolkov et al. 2025). We also recovered reads assigned to New World taxa, which cannot plausibly occur in our samples given their biogeographic and temporal contexts. Such implausible assignments are a well‐documented consequence of competitive k‐mer–based classification and can arise from at least two sources. Reference genomes frequently contain contaminant sequences—Steinegger and Salzberg (2020) identified more than 2 million contaminated entries in GenBank—and any contamination not removed by our pre‐filtering against the GTDB and RefSeq databases can misdirect reads towards the taxon whose reference harbors the contaminant rather than their true source. In addition, genomic regions conserved among related lineages, including geographically disjunct ones, can cause reads to be assigned to a taxon that is not genuinely present, particularly when that taxon is more completely represented in the database than the true source. In addition, there is a general difficulty in distinguishing dietary taxa from environmental contamination. While some of the taxa detected in dental calculus samples from this study could plausibly represent dietary items (*Equus*, *Lepus*, *Brassica*, *Cornus*, *Prunus*, *Triticum*, and *Vitis*), others are likely to be naturally occurring around the sites and the cemeteries both during and after the lifetime of the studied individuals. All eukaryotic taxa that passed our filters were also detected in soil samples. Therefore, even after decontamination, we cannot exclude the possibility that the individuals came into contact with the eukaryotic species only after death. At the same time, it is not surprising to detect signals of cultivated plants and farmed animals in soil samples, given that they might have been planted and cared for in the same place where humans lived and consumed them. In summary, disentangling dietary and environmental signals from dental calculus remains a considerable challenge, which can likely only be addressed by consulting additional sources of information on food consumption in the past (such as written records, organic residues in ceramic vessels and artistic depictions).

Because dental calculus also contains some host reads, we attempted to infer the provenance of the individuals under study. This is of particular interest in the context of the Roman Empire, which has been associated with high population mobility. The few studies available for Italy during the Roman period depict a cosmopolitan society characterized by various genetic ancestries (Antonio et al. 2019; Ravasini et al. 2024). In Lucus Feroniae, historical sources point to a diverse population, including war veterans, slaves, and freemen from various regions of the Empire. However, only 11 individuals yielded sufficient data to attempt ancestry inferences, and all of them were consistent with close genetic similarity to present‐day Tuscany/Iberia. Overall, without applying a targeted enrichment protocol (Ozga et al. 2016), the number of endogenous reads was too low for meaningful population‐level analyses. These findings are in line with previous studies highlighting the limited presence of host DNA in human dental calculus samples (Mann et al. 2018), in contrast to the considerably higher proportion of host DNA in dental calculus from non‐human mammals (Brealey et al. 2020; Moraitou et al. 2022, 2025).

## 5 Conclusions

By studying the oral microbiome of individuals buried in three different cemeteries spanning from I to VIII centuries CE, we detected differences between both contemporary and asynchronous populations, which could result from differences in subsistence practices, access to resources, and general health status. To the best of our knowledge, we present the first record of differences in oral microbiome taxonomic and functional composition in human societies that is associated with social class, possibly as a result of trade, local farming practices, and population density. Although we cannot establish a causal relationship between these factors and the oral microbiome, particularly as dietary characterization from dental calculus remains insufficient, our findings suggest an exciting possibility that “lifestyle indicators” could be detectable from preserved microbial communities, contributing to the extended study of past societies. Further analyses are needed to better understand dietary choices at the three sites and how they might have impacted oral microbial communities. Overall, we find a good correspondence between the results obtained from dental calculus and previous palaeopathological, morphological, and isotopic studies, thereby expanding our knowledge of past human societies, particularly for previously underexplored groups.

## Author Contributions

**Martina Farese:** data curation, investigation, formal analysis, writing – original draft, writing – review and editing, visualization, validation, funding acquisition. **Tom van der Valk:** methodology, formal analysis, writing – review and editing, writing – original draft. **Chenyu Jin:** methodology, formal analysis, writing – review and editing, writing – original draft. **Adrian Forsythe:** methodology, investigation, writing – review and editing. **Giorgio Manzi:** resources. **Ileana Micarelli:** resources. **Laura Parducci:** writing – review and editing, resources. **Katerina Guschanski:** conceptualization, methodology, supervision, funding acquisition, project administration, writing – original draft, writing – review and editing, resources. **Markella Moraitou:** investigation, formal analysis, writing – review and editing, writing – original draft, visualization, methodology, validation. **Mary Anne Tafuri:** resources, conceptualization, writing – review and editing, supervision.

## Funding

This work was supported by the Bertil Lundman Foundation for Anthropological Studies (Swedish Phytogeographical Society) and the Italian Ministry of University and Research (MUR) (DOT1326JZS).

## Conflicts of Interest

The authors declare no conflicts of interest.

## Supporting information


**Figure S1:** Principal coordinate analysis (PCoA) ordination based on the taxonomic composition of dental calculus (in green), extraction and library blanks (in dark and light gray, respectively) and soil (in brown) following decontamination.
**Figure S2:** Abundance ranks of HOMD (Human Oral Microbiome Database) taxa found in extraction blanks. Orange dots show the abundance rank of a certain taxon in a blank (1 = most abundant, 2 = second most abundant, etc.), and black dots show the abundance rank in dental calculus samples from the same batch and from all other batches (grouped under “batch other”).
**Figure S3:** Damage pattern plots ordered alphabetically by microbial species. The mode value of fragment length is also noted. The figure continues on the next page.
**Figure S4:** Rarefaction curves showing observed microbial diversity when subsampling each sample to 100–10,000 reads. (A) Only healthy teeth, colored by cemetery of origin. (B) All samples, colored by health state.
**Figure S5:** Observed species richness (top row) and Shannon evenness index (bottom row) for different cemeteries and tooth health states. Asterisks indicate samples that were excluded from alpha diversity tests with ANOVA because they had fewer than 4000 taxonomically classified reads after filtering and decontamination. “Healthy in caries mouth” corresponds to dental calculus microbiome from healthy teeth sampled in an oral cavity with presence of caries elsewhere, whereas “Caries” samples were obtained directly from the caries lesions.
**Figure S6:** CLR‐normalized abundances of caries‐associated taxa (according to Zhang et al. 2022 and HOMD, species names amended according to NCBI taxonomy) and taxa putatively associated with endocarditis (according to Cahill and Prendergast 2016 and HOMD). *Streptococcus gordonii* was placed under the “other” category because, while being a part of the healthy oral microflora, it has also been associated with various conditions, like caries, infective endocarditis and aphthous ulcerations. Gray color indicates a taxon was not detected in a given sample. The highlighted samples are from individuals with caries (yellow: healthy tooth in individual with caries, red: tooth with caries).
**Figure S7:** Log10‐transformed ratio of the total abundance of early colonizers (*Streptococcus* and *Actinomyces*) and late colonizers of dental plaque (*Methanobrevibacter* only, as the red complex bacteria were absent). The background color indicates tooth health status (blue: individual without caries, yellow: healthy tooth in individual with caries, red: tooth with caries). A value of 0 indicates that early and late colonizers have the same total abundance in a given sample, positive values indicate that *Methanobrevibacter* is more abundant than early colonizers, whereas negative values indicate that it is less abundant.
**Figure S8:** Principal coordinate analysis (PCoA) ordination based on the GO term composition (only biological processes) of dental calculus (in green) and soil (in brown). The 10 dental calculus samples to the left of the dotted line were considered poorly preserved, as they clustered closer to the soil samples, and were consequently excluded from downstream functional analysis.

**Table S1:** List of dental calculus samples, soil samples and blanks used in the metagenomic analysis, with provenance, sampling and processing information.
**Table S2:** Kraken2 table, showing raw abundances of taxa across samples.
**Table S3:** Microbial genomes used as mapping references to assess deamination damage.
**Table S4:** Information and NCBI accession numbers for the reference mammal, fish and plant genomes used for dietary analysis.
**Table S5:** List of retained microbial taxa and their relative abundance for each sample.
**Table S6:** List of putative pathogens and whether they were identified in the dataset (for abundances see Figure S6).
**Table S7:** Abundances of GO terms associated with biological processes for each sample.
**Table S8:** List of eukaryotic taxa (plants, mammals and fish) used for dietary analysis. The average relative abundance of each taxon is presented for dental calculus, soil and blanks, as well as for the dental calculus/blanks and the dental calculus/soil comparisons.
**Table S9:** List of retained mammal, fish and plant taxa for the dietary analysis, after decontamination procedures. The average relative abundance of each retained taxon is presented for dental calculus, soil and blanks.
**Table S10:** Number of reads or sites mapped for the dietary analysis to each taxon (mammals, fish and plants) for each sample (dental calculus, soil and blanks).
**Table S11:** Molecular sex identification results for the 11 dental calculus and 3 soil samples with ≥ 1000 reads mapping to the X chromosome and the autosomes.
**Table S12:** Genetic affinity results for the 11 samples with ≥ 10,000 SNPs of the reference panel covered by at least one sequencing read.

## Data Availability

The data used in this study are available in the article and its Supporting Information. The raw sequencing data (FASTQ files) used in this study have been deposited in the ENA repository under accession number PRJEB106785. The scripts used for this study are available at https://github.com/markella‐moraitou/Roman_oral_microbiomes and archived through Zenodo under the DOI 10.5281/zenodo.19666869.
