# Supplementary material for: Lifestyle Impacts the Oral Microbiome of Classical and Post‐Classical Societies in Italy
Source: Am J Biol Anthropol. 2026 Sep 8;191(1):e70357. doi: 10.1002/ajpa.70357 (PMC13554472; doi:10.1002/ajpa.70357)
Supplement: Supplementary file 1 [file AJPA-191-e70357-s001.docx]

Supplementary Figures to “**Lifestyle impacts the oral microbiome of Classical and Post-classical societies in Italy**”

**Figure S1.** Principal Coordinate Analysis (PCoA) ordination based on the taxonomic composition of dental calculus (in green), extraction and library blanks (in dark and light grey, respectively) and soil (in brown) following decontamination.

**Figure S2.** Abundance ranks of HOMD (Human Oral Microbiome Database) taxa found in extraction blanks. Orange dots show the abundance rank of a certain taxon in a blank (1=most abundant, 2=second most abundant, etc.), and black dots show the abundance rank in dental calculus samples from the same batch and from all other batches (grouped under “batch other”).

**Figure S3.** Damage pattern plots ordered alphabetically by microbial species. The mode value of fragment length is also noted. The figure continues on the next page.

**Figure S3 (continued from previous page).** Damage pattern plots ordered alphabetically by microbial species. The mode value of fragment length is also noted. The figure continues on the next page.

**Figure S3 (continued from previous page).** Damage pattern plots ordered alphabetically by microbial species. The mode value of fragment length is also noted.

**Figure S4.** Rarefaction curves showing observed microbial diversity when subsampling each sample to 100-10,000 reads. **A.** Only healthy teeth, coloured by cemetery of origin. **B.** All samples, coloured by health state.

**Figure S5**. Observed species richness (top row) and Shannon evenness index (bottom row) for different cemeteries and tooth health states. Asterisks indicate samples that were excluded from alpha diversity tests with ANOVA because they had fewer than 4,000 taxonomically classified reads after filtering and decontamination. “Healthy in caries mouth” corresponds to dental calculus microbiome from healthy teeth sampled in an oral cavity with presence of caries elsewhere, whereas “Caries” samples were obtained directly from the caries lesions.

**Figure S6**. CLR-normalised abundances of caries-associated taxa (according to Zhang et al., 2022 and HOMD, species names amended according to NCBI taxonomy) and taxa putatively associated with endocarditis (according to Cahill and Prendergast 2016 and HOMD). *Streptococcus gordonii* was placed under the ‘other’ category because, while being a part of the healthy oral microflora, it has also been associated with various conditions, like caries, infective endocarditis and aphthous ulcerations. Grey colour indicates a taxon was not detected in a given sample. The highlighted samples are from individuals with caries (yellow: healthy tooth in individual with caries, red: tooth with caries).

**Figure S7.** Log10-transformed ratio of the total abundance of early colonisers (*Streptococcus* and *Actinomyces*) and late colonisers of dental plaque *(Methanobrevibacter* only, as the red complex bacteria were absent). The background colour indicates tooth health status (blue: individual without caries, yellow: healthy tooth in individual with caries, red: tooth with caries). A value of 0 indicates that early and late colonisers have the same total abundance in a given sample, positive values indicate that *Methanobrevibacter* is more abundant than early colonisers, whereas negative values indicate that it is less abundant.

**Figure S8**. Principal Coordinate Analysis (PCoA) ordination based on the GO term composition (only biological processes) of dental calculus (in green) and soil (in brown). The ten dental calculus samples to the left of the dotted line were considered poorly preserved, as they clustered closer to the soil samples, and were consequently excluded from downstream functional analysis.
